# Isolation and characterization of novel reassortant H6N1 avian influenza viruses from chickens in Eastern China

**DOI:** 10.1186/s12985-018-1063-y

**Published:** 2018-10-24

**Authors:** Haibo Wu, Fan Yang, Fumin Liu, Rufeng Lu, Xiuming Peng, Bin Chen, Hangping Yao, Nanping Wu

**Affiliations:** 1 State Key Laboratory for Diagnosis and Treatment of Infectious Diseases, Collaborative Innovation Center for Diagnosis and Treatment of Infectious Diseases, the First Affiliated Hospital, School of Medicine, Zhejiang University, 79 Qingchun Road, Zhejiang, 310003 Hangzhou China; 2 Department of Emergency, The First Affiliated Hospital of Zhejiang Chinese Medical University, Hangzhou, China

**Keywords:** Avian influenza viruses, Subtype H6N1, Chickens, Reassortant, Eastern China

## Abstract

**Background:**

The H6N1 subtype of avian influenza viruses (AIVs) can infect people with an influenza-like illness; the H6N1 viruses possess the ability for zoonotic transmission from avians into mammals, and possibly pose a threat to human health.

**Methods:**

In 2017, live poultry markets (LPMs) in Zhejiang Province were surveyed for AIVs. To better understand the genetic relationships between these strains from Eastern China and other AIVs, all gene segments of these strains were sequenced and compared with sequences available in GenBank. In this study, we analyzed the receptor-binding specificity, antigenic characteristics, and pathogenicity of these two H6N1 viruses.

**Results:**

In 2017, two H6N1 AIVs were isolated from chickens during surveillance for AIVs in LPMs in Eastern China. Phylogenetic analysis showed that these strains shared genetic characteristics from H6, H10, H1, and H4 AIVs found in ducks and wild birds in East Asia. These AIV strains were able to replicate in mice without prior adaptation.

**Conclusions:**

In this study, we report the discovery of new strains of H6N1 viruses from chickens with novel gene reassortments. Our results suggest that these chickens play an important role generating novel reassortments in AIVs, and emphasize the need for continued surveillance of AIV strains circulating in poultry.

**Electronic supplementary material:**

The online version of this article (10.1186/s12985-018-1063-y) contains supplementary material, which is available to authorized users.

## Background

Influenza A viruses are classified into 18 hemagglutinin (HA) and 11 neuraminidase (NA) subtypes, based on the antigenic properties of the HA and NA glycoproteins [1, 2]. Aquatic birds are considered a natural reservoir for avian influenza virus (AIV) [3]. Birds infected with AIVs, including poultry and wild birds, do not usually display clinical symptoms; however, they provide an environment for the reassortment of low pathogenic AIVs, which can serve as progenitors of highly pathogenic AIVs [4, 5]. The adaptation of AIVs to receptors in poultry can enhance the potential for avian-to-human transmission of AIVs; populations of terrestrial poultry species, especially chickens and quails, play an important role in expanding the host of influenza viruses [6–8].

The H6 subtypes of AIVs were first isolated from a turkey in 1965 in the United States and were subsequently identified in wild migratory birds [9–13]. H6 AIVs have infected ducks and chickens, and circulate in live poultry markets (LPMs) in China [14–19]. In China, serological evidence has demonstrated human infection with H6 AIVs, as well as infection in other mammals [20]. Previous studies in Taiwan showed that the H6N1 subtype of low pathogenic AIVs can infect humans with an influenza-like illness [21, 22]. In 2014, an H6N1 virus was isolated from dogs in Taiwan; molecular analysis indicated that this isolate was closely related to the human H6N1 virus circulating in Taiwan, and harbored the E627K substitution in the polymerase basic protein 2 (PB2) [23]. In addition, the human-infecting H6N1 virus possesses the ability to cross the species barrier to infect other mammals [23, 24], these results also indicated that E190V and G228S mutations of HA are important to acquire the human receptor-binding capacity, and the P186L mutation of HA could reduce the avian receptor-binding capacity [25]. The H6 viruses possess similar internal genes to the human H5N1 and H9N2 viruses [17, 26]. These data indicate that the H6 AIVs pose a threat to human health, and emphasize the need for continued surveillance of the H6 AIVs circulating in poultry.

In 2017, during surveillance of poultry for AIVs in Zhejiang Province, Eastern China, two H6N1 viruses, named A/chicken/Zhejiang/1664/2017(H6N1) and A/chicken/Zhejiang/1667/2017 (H6N1) (ZJ-1664 and ZJ-1667, respectively), were isolated from apparently healthy chickens. To better understand the genetic relationships between these strains from Eastern China and other AIVs, all gene segments of these strains were sequenced and compared with sequences available in GenBank. These findings indicate that continued surveillance of H6N1 AIVs in poultry should be used as an early warning system for potential avian influenza outbreaks.

## Methods

### Ethics statement

Female 6-week-old pathogen-free BALB/c mice were purchased from Shanghai Laboratory Animal Center, Chinese Academy of Sciences, Shanghai, China, and housed in filter-top cages. The animal studies in this research were conducted in accordance with guidelines of animal welfare of World Organization for Animal Health. The protocols for mouse and embryonated chicken egg experiments were approved by the Ethics Committee of the First Affiliated Hospital, School of Medicine, Zhejiang University (permit number. 2015–015).

### Virus isolation

Cloacal swabs (*n* = 72) were collected from apparently healthy poultry in a LPM in Hangzhou, Zhejiang Province, Eastern China, on January 6, 2017. Each sample was inoculated into the allantoic cavities of 9-day-old specific pathogen-free embryonated eggs as described previously [27, 28]. After incubation at 37 °C for 72 h, the allantoic fluid was harvested and viral titers were determined by hemagglutination assay using a standard method, as described previously [28].

### RNA extraction and PCR amplification

RNA was extracted from HA-positive allantoic fluid samples using TRIzol (Life Technologies, USA) according to the manufacturer’s instructions. Reverse transcription was performed using the Uni12 primer: 5′–AGCAAAAGCAGG–3′. Reverse transcriptase-polymerase chain reaction (RT-PCR) was conducted using a One-Step RNA PCR Kit (TaKaRa, China) as described previously [28]. All segments were amplified with previously described segment–specific primers [29]. The PCR products were purified using an Agarose Gel DNA Fragment Recovery Kit Ver. 2.0 (TaKaRa).

### Sequencing and phylogenetic analysis

Amplified PCR fragments were sequenced using a Big Dye Terminator V.3.0 Cycle Sequencing Ready Reaction kit (ABI, Foster City, CA, USA) according to the manufacturer’s instructions. All eight gene segments of the AIV field isolates were sequenced, including PB2, polymerase basic protein 1 (PB1), polymerase acidic protein (PA), HA, nucleocapsid protein (NP), NA, matrix protein (M), and nonstructural protein (NS); sequences were compared with those from reference viruses. The classical reference viruses were selected based on previous reports [14, 18, 26], and the reference sequences of the strains used in this study were obtained from the Influenza Virus Resource (https://www.ncbi.nlm.nih.gov/genomes/FLU/Database/nph-select.cgi?go=database). Sequences were analyzed using BioEdit DNA software version 7.0.9.0. Phylogenetic trees were constructed using molecular evolutionary genetics analysis (MEGA) software version 6.0, applying the maximum likelihood method and the Tamura–Nei model with bootstrap analysis (1000 replicates) as described previously [28, 30]. The nucleotide sequences were deposited into GenBank under the accession numbers: MG063430–45.

### Prediction of glycosylation sites

The NetNGlyc 1.0 server was used to predict potential *N*-linked glycosylation sites (defined as Asn-X-Ser/Thr, where X is any amino acid except Asp or Pro); a threshold value > 0.5 for the average potential score suggests glycosylation [31, 32].

### Receptor-binding analysis

The α-2,3-specific sialidase-treated red blood cell (RBC) hemmaglutination assay is commonly employed to screen receptor specificity [33, 34]. The receptor binding preference of the H6N1 AIVs was determined by performing hemagglutination assays on receptor-specific RBCs, including normal chicken RBCs (containing both α-2,6 and α-2,3 receptor), α-2,3-specific sialidase-treated chicken RBCs (containing only α-2,6 receptor after treatment), and sheep RBCs (mainly expressing α-2,3 receptor). The α-2,6 receptor-specific RBCs were prepared by treating chicken RBCs with α-2,3 specific neuraminidase (NEB, MA) as described previously [35–37]. Briefly, 10% RBC in 1 mL were treated with 1000 IU α-2, 3-specific neuraminidase for 1 h at 37 °C, and washed three times with PBS. The final working solution for the hemagglutination assay was 0.5% RBCs in PBS. To determine the specific receptors on the treated and untreated RBCs, the avian influenza virus A/chicken/Zhejiang/329/2011(H9N2) and human influenza virus A/Puerto Rico/8/1934(H1N1) strains were included in the receptor binding assay as controls. Hemmaglutination assays were performed in 96‘V’-well microtiter plates by incubating equal volumes (50 μL) of a two-fold serial dilution of virus with each RBC type. The HA titer was defined as the reciprocal of the highest virus dilution resulting in the hemagglutination of RBCs.

### Antigenic analysis

The antigenic characteristics of these H6N1 AIVs were analyzed with a hemagglutination inhibition (HI) test, as described previously [38]. Mouse antisera against A/chicken/Zhejiang/727031/2014 (H6N2), A/duck/Zhejiang/727038/2014 (H6N2), A/chicken /Zhejiang/727001/2014 (H6N6), A/chicken/Zhejiang/727028/2014 (H6N6), A/chicken /Zhejiang/110102/2015 (H6N6), A/chicken/Zhejiang/C1/2013(H9N2), and A/goose/Zhejiang/112071/2014(H5N1) were provided by our laboratory, the H9N2 and H5N1 viruses were included as controls. The mouse antisera used for HI assay have been treated by receptor destroying enzyme (RDE, Denka Seiken, Japan). These viruses were all recently isolated from LPMs in Zhejiang Province, China, as described previously [18, 19].

### Viral growth kinetics

Confluent MDCK or A549 cells were infected with ZJ-1664 and ZJ-1667 strains at a multiplicity of infection (MOI) of 0.1, overlaid with serum-free DMEM containing 2 mg/mL TPCK-trypsin (Sigma-Aldrich, USA) and incubated at 37 °C as described previously [39–41]. Cell supernatants were harvested every 12 h until 72 h postinoculation and titrated in embryonated chicken eggs by the Reed and Muench method [42].

### Animal studies

To evaluate the replicative ability of the ZJ-1664 and ZJ-1667 strains in a mammalian host, fifteen 6-week-old female BALB/c mice were infected intranasally with 10^6.0^ EID50 per 1 ml of each virus; three mice were sacrificed at 3, 6, and 9 days post-inoculation. Lungs, brains, hearts, livers, kidneys, and spleens were collected for viral titer analysis in embryonated chicken eggs by the method of Reed and Muench [42]. The survival of the six remaining mice was observed for 14 days following inoculation.

### Histology and immunohistology of mouse lung sections

Lung tissues from virus–inoculated mice were fixed in 10% neutral buffered formalin for at least 24 h before processing. The tissues were embedded in paraffin by standard tissue processing procedures. Sections “Discussion” μm thick were cut and fixed on glass slides. Standard hematoxylin and eosin (H&E) staining was carried out. Immunohistochemical staining to detect nucleoprotein antigens in the lungs was performed using a monoclonal antibody against the influenza A virus nucleoprotein (1:100 dilution). The sections were washed 3 times with PBS and then incubated with HRP–conjugated goat anti–mouse secondary antibody (1:3000 dilutions, Sigma). The sections were developed with 3–3′ diaminobenzidine and examined with a light microscope, as described previously [43].

## Results

### Virus isolation

Three strains of AIVs were isolated from 72 cloacal swab samples collected from chickens in a LPM in the Yuhang district of Hangzhou City, the capital of Zhejiang Province, including one H9N2 and two H6N1 strains, ZJ-1664 and ZJ-1667.

### Phylogenetic analysis of the ZJ-1664 and ZJ-1667 H6N1 strains

**Fig. 1:**
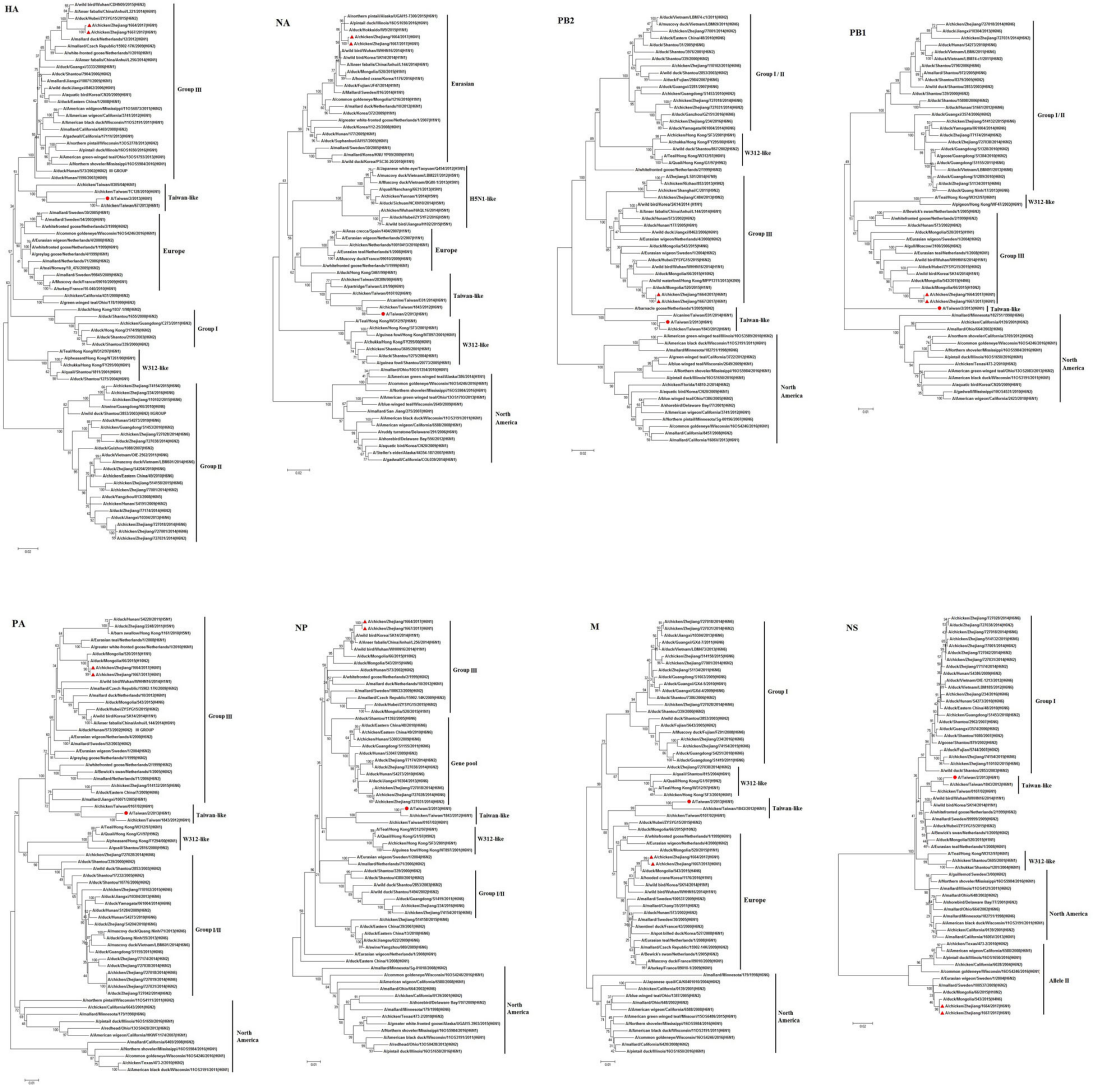
Phylogenetic trees of HA (positions 1–1701), NA (positions 1–1410), PB2 (positions 1–2280), PB1 (positions 1–2274), PA (positions 1–2151), NP (positions 1–1497), M (positions 1–982), and NS (positions 1–838) segments from the novel reassortant H6N1 avian influenza viruses, ZJ-1664 and ZJ-1667. The tree was created by the maximum likelihood method and bootstrapped with 1000 replicates using the MEGA6 software version 6.0. The H6N1 viruses characterized are indicated by a triangle in red, and the H6N1 influenza virus responsible for human infection in 2013 is indicated by a dot in red. The scale bar represents the distance unit between sequence pairs

Phylogenetic analysis of all eight viral segments, PB2, PB1, PA, HA, NP, NA, M, and NS, showed that the ZJ-1664 and ZJ-1667 strains clustered in the AIV Eurasian lineage, Fig. 1. According to the HA genes, the H6 viruses from China are divided into three lineages: group I, group II (including W312-like), and group III [14, 18, 26]. Phylogenetic analysis of the HA genes from the ZJ-1664 and ZJ-1667 isolates indicated that these viruses were closely related to the group III viruses that circulated in Eurasian regions from 2009 to 2015, represented by the A/duck/Hunan/573/2002 (H6N2) strain. The phylogeny of the HA genes also indicated that these viruses have a different lineage from the H6N1 virus responsible for human infection in Taiwan in 2013 [21]. We previously reported that the HA genes of H6 AIVs found in the Zhejiang Province (H6N2 and H6N6) belong to the group II viruses, represented by the A/wild duck/Shantou/2853/2003 (H6N2) strain [18, 19]. These results suggest that at least two different genetic groups of the H6 viruses have spread through the Zhejiang Province in recent years.

Regarding the phylogeny of the NA gene, the ZJ-1664 and ZJ-1667 H6N1 viruses were most closely related to AIVs found in wild birds in Asia between 2014 and 2016. The sequence homologies of the HA and HA genes from the ZJ-1664 and ZJ-1667 strains were similar to the AIV reference strains, and the ZJ-1664 isolate was selected for in depth analysis. The percent sequence homology for each gene segment from ZJ-1664 compared to the closest genetic relative is shown in Table 1.

**Table 1:** Sequence homology of the whole genome of the ZJ-1664 H6N1 avian influenza virus compared to influenza virus nucleotide sequences available in the GenBank database

Segment	Position	Virus with the highest percentage of nucleotide identity	Genbank accession number	Homology (%)	Lineage
PB2	1–2280	A/duck/Mongolia/520/2015(H1N1)	LC121393	99	group III
PB1	1–2274	A/duck/Mongolia/66/2015(H10N2)	LC108118	98	group III
PA	1–2151	A/duck/Mongolia/66/2015(H10N2)	LC108119	99	group III
HA	1–1701	A/duck/Hubei/ZYSYG15/2015(H6N2)	KY415608	98	group III
NP	1–1497	A/wild bird/Korea/SK14/2014(H1N1)	KX066872	99	group III
NA	1–1410	A/wild bird/Wuhan/WHHN16/2014(H1N1)	KU143337	99	Eurasian
M	1–959	A/duck/Mongolia/543/2015(H4N6)	LC121415	99	Europe
NS	1–838	A/duck/Mongolia/543/2015(H4N6)	LC121416	99	Allele II

**Fig. 2:**
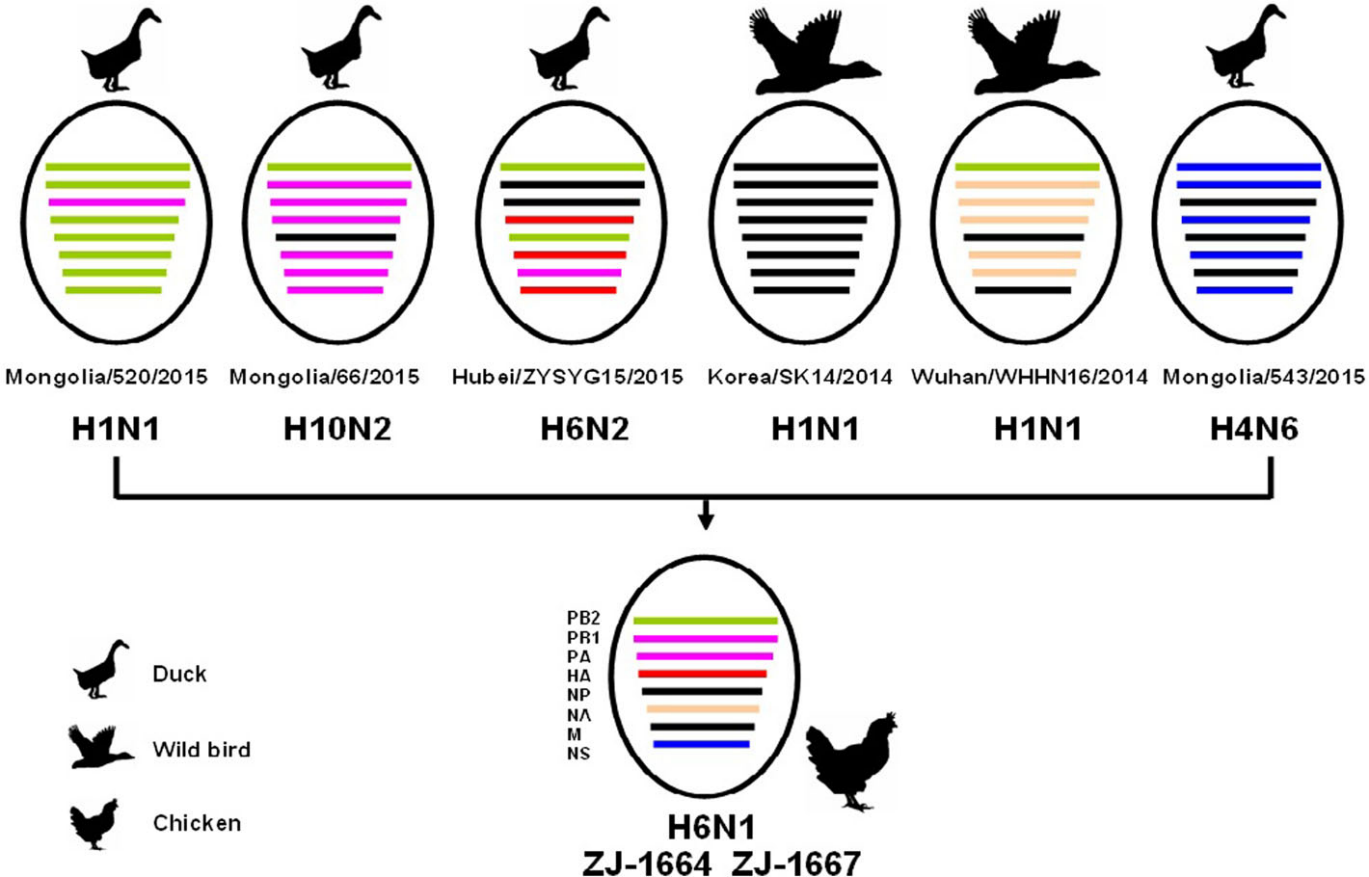
A simplified schematic showing the putative genomic composition of the novel reassortant H6N1 avian influenza viruses, ZJ-1664 and ZJ-1667. The eight gene segments (from top to bottom) in each virus are polymerase basic protein 2 (PB2), polymerase basic protein 1(PB1), polymerase acidic protein (PA), hemagglutinin (HA), nucleocapsid protein (NP), neuraminidase (NA), matrix protein (M), and nonstructural protein (NS). Each color represents a separate virus background. Mongolia/520/2015, A/duck/Mongolia/520/2015(H1N1); Mongolia/66/2015, A/duck/Mongolia/66/2015(H10N2); Hubei/ZYSYG15/2015, A/duck/Hubei /ZYSYG15/2015(H6N2); Korea/SK14/2014, A/wild bird/Korea/SK14/2014(H1N1); Wuhan/WHHN16/2014, A/wild bird/Wuhan/WHHN16/2014(H1N1); Mongolia/543/2015, A/duck/Mongolia/543/2015(H4N6). The illustration is based on the nucleotide-distance comparison and phylogenetic analysis

Homologous comparison to the GenBank reference database using BLAST showed that the HA and NA genes from ZJ-1664 had the highest similarity to the HA and NA genes from A/duck/Hubei/ZYSYG15/2015 (H6N2) and A/wild bird/Wuhan/WHHN16/2014 (H1N1) strains, respectively. The PB2, PB1, PA, NP, M, and NS genes from ZJ-1664 shared the highest similarity with H1, H10, and H4 isolates, which were isolated from birds throughout Eastern Asia between 2014 and 2015 (Fig. 1, Table 1). Previous reports showed that the HA genes of H6 AIVs (including H6N1, H6N2, H6N5, H6N6, and H6N8) were cocirculated in domestic ducks in Eastern China, and these H6 viruses all underwent frequent reassortment with multiple virus subtypes [15, 16, 18, 19]. Based on the phylogenetic and homology analyses, ZJ-1664 is a result of viral reassortment from H6, H1, H10, and H4 AIV subtypes from poultry and wild bird in Eastern Asia (Fig. 2). These results are also indicative of the active evolution of H6 AIVs in Eastern China.

### Molecular characterization of the ZJ-1664 and ZJ-1667 H6N1 strains

Based on the deduced amino acid sequences of the HA proteins, the cleavage site pattern PQIETR/GL occurs in the HA from both the ZJ-1664 and ZJ-1667 H6N1 viruses. The presence of a monobasic cleavage site indicates that these strains are low pathogenic AIVs [44]. The amino acids at receptor binding sites were well conserved (Table 2). The receptor binding sites of ZJ-1664 and ZJ-1667, Q226 and G228 (H3 numbering), are similar to other H6 AIVs, suggesting that these strains may preferentially bind α-2,3 linked sialic acid receptors, which are predominant in avian species [45]. The P186L, E (or D)190 V and G228S substitutions have been reported to favor the affinity of influenza viruses for human type receptors [21, 46]; these substitutions were not observed in ZJ-1664 or ZJ-1667, Additional file 1: Figure S1.

**Table 2:** Conservation analysis of amino acids at the cleavage site and receptor-binding sites in the HA protein of the ZJ-1664 and ZJ-1667 H6N1 strains and reference H6 viruses

H3 numbering system	N-linked glycosylation sites in HA	HA cleavage	Receptor-binding site							Left edge of receptor-binding site	Right edge of receptor-binding site
		**320–329**	**98**	**153**	**155**	**183**	**190**	**194**	**195**	**220–229**	**134–138**
**H6 position**		**339–344**	**107**	**166**	**168**	**197**	**204**	**208**	**209**	**234–243**	**146–150**
ZJ-1664(H6N1)	5	PQIETR	Y	W	I	H	E	L	Y	RPAVNGQRGR	GV**TK**A
ZJ-1667(H6N1)	6	PQIETR	Y	W	I	H	E	L	Y	RPAVNGQRGR	GV**TK**A
A/chicken/Zhejiang/727031/2014(H6N2)	5	PQIETR	Y	W	**V**	H	E	L	Y	RPAVNGQRGR	GVSSA
A/duck/Zhejiang/727038/2014(H6N2)	6	PQIETR	Y	W	I	H	E	L	Y	RPAVN**A**QRGR	GVSSA
A/chicken/Zhejiang/727001/2014(H6N6)	5	PQIETR	Y	W	**V**	H	E	L	Y	RPAVNGQRGR	GVSSA
A/chicken/Zhejiang/727028/2014(H6N6)	6	PQIETR	Y	W	I	H	E	L	Y	**K**PAVNGQRGR	GVSSA
A/chicken/Zhejiang/110102/2015(H6N6)	5	PQIETR	Y	W	I	H	E	L	Y	RP**V**VNGQRGR	GV**T**S**S**
A/duck/Hubei/ZYSYG15/2015(H6N2)	6	PQIETR	Y	W	I	H	E	L	Y	RPAVNGQRGR	GV**TK**A
A/chicken/Hunan/S4191/2009(H6N2)	5	PQIETR	Y	W	**V**	H	E	L	Y	RPAVNGQRGR	GVSSA
A/duck/Shantou/2195/2003(H6N2)	6	PQIETR	Y	W	I	H	E	L	Y	RPAVNGQRGR	GVSSA
A/wild duck/Shantou/2853/2003(H6N2)	5	PQIETR	Y	W	I	H	E	L	Y	RPAVNGQRGR	GV**T**S**S**
A/duck/Hunan/573/2002(H6N2)	5	PQIETR	Y	W	I	H	E	L	Y	RPAVNGQRGR	GV**TK**A
A/duck/Eastern China/48/2010(H6N6)	5	PQIETR	Y	W	I	H	E	L	Y	RPAVNGQRGR	GVSSA
A/duck/Jiangxi/5178/2007(H6N2)	5	PQIETR	Y	W	I	H	E	L	Y	RPAVNGQRGR	GVSSA
A/duck/Zhejiang/S4204/2010(H6N6)	5	PQIETR	Y	W	I	H	E	L	Y	RPAVNGQRGR	GVSSA
A/swine/Guangdong/K6/2010(H6N6)	5	PQIETR	Y	W	I	H	E	L	Y	RP**V**VNGQR**S**R	GV**T**S**S**
A/muscovyduck/Vietnam/LBM601/2014(H6N6)	5	PQIETR	Y	W	I	H	E	L	Y	RPAVNGQRGR	GVSSA
A/mallard/Ohio/664/2002(H6N6)	5	PQ**VK**TR	Y	W	I	H	E	L	Y	RP**PA**NGQRGR	GVSSA
A/Teal/Hong Kong/W312/97(H6N1)	6	PQIETR	Y	W	I	H	E	L	Y	RPAVNGQRGR	G**TTRS**
A/Taiwan/2/2013(H6N1)	6	PQI**A**TR	Y	W	**V**	H	**V**	L	Y	RPAVNGQR**S**R	GV**TN**A

Residues in **bold** indicate differences from the consensus alignment

No substitutions associated with resistance to NA inhibitors (oseltamivir; His275Tyr substitution) or amantadines were observed in the NA [47] and M2 proteins (Val27Ala and Ser31Asn substitutions) [48] in ZJ-1664 or ZJ-1667. The PB2 protein substitution Glu627Lys is associated with the pathogenicity of AIVs in mice [49], and the Thr271Ala substitution plays a key role in enhanced polymerase activity of influenza viruses in mammalian host cells [50]; neither of these substitutions was observed in the PB2 of ZJ-1664 or ZJ-1667.

### Prediction of glycosylation sites in the ZJ-1664 and ZJ-1667 H6N1 strains

*N*-linked glycosylation of HA is associated with virulence, alterations in HA antigenicity, and viral affinity for the influenza virus receptor [51, 52]. We predicted potential *N*-linked glycosylation sites in the HA and NA of the ZJ-1664 and ZJ-1667 strains by using the NetNGlyc 1.0 server, which predicted five (or six) potential *N*-linked glycosylation sites in HA (HA1, 27, (38), 182 and 306; HA2, 498 and 557, Table 2) and seven in NA (50, 58, 63, 68, 88, 146, and 235). These H6N1 strains have different N-linked glycosylation sites in HA suggesting that they probably possess different biological characteristics.

### Receptor-binding analysis of the ZJ-1664 and ZJ-1667 H6N1 strains

Receptor-binding specificity of HA is a key determinant for viral host range [53]. We analyzed the receptor-binding specificity of the H6N1 viruses using HA assays with sialidase-treated RBCs, which have only α-2,6 receptor, normal chicken RBCs (containing both α-2,6 and α-2,3 receptors), and sheep RBCs (mainly expressing the α-2,3 receptor). Analysis of the viral titers revealed that ZJ-1664 and ZJ-1667 agglutinated all three types of RBCs, indicating that they possess specificity toward both the avian and human receptors (Table 3).

**Table 3:** Virus receptor binding specificity of the avian H6N1, avian H9N2 and human H1N1 influenza viruses

Virus	HA titers with normal chicken RBCs	HA titers with α-2,3 specific-neuraminidase-treated chicken RBCs	HA titers with sheep RBCs	Receptor specificity
ZJ-1664(H6N1)	256	32	256	α2,3-SA and α2,6-SA binding
ZJ-1667(H6N1)	256	32	256	α2,3-SA and α2,6-SA binding
A/chicken/Zhejiang/329/2011(H9N2)	256	< 2	256	α2,3-SA binding
A/Puerto Rico/8/1934(H1N1)	256	256	< 2	α2,6-SA binding

**Note:** The HA titer was defined as the reciprocal of the highest virus dilution resulting in the hemagglutination of RBCs. “α2,3-SA” and “α2,6-SA” indicate “α-2,3-linked sialic acids” (avian receptor) and “α-2,6-linked sialic acids” (human receptor), respectively

### Antigenic analysis of the ZJ-1664 and ZJ-1667 H6N1 strains

Antigenic characterization is an important tool in the selection of strains to maintain updated influenza vaccinations [54]. To evaluate the antigenic characteristics of ZJ-1664 and ZJ-1667, we used the HI assay to compare cross–reactivity with other H6 viruses (H6N2 and H6N6), which were recently isolated from chickens and ducks in Zhejiang Province (Table 4). The ZJ-1664 and ZJ-1667 strains did not react well with the other Zhejiang H6N2 and H6N6 AIVs belonging to group II (HA gene).

**Table 4:** Antigenic analysis of the ZJ-1664 and ZJ-1667 H6N1 influenza viruses according to the hemagglutination inhibition test

Polyclonal antisera titers to	Reassortant H6N1 virus	
	ZJ-1664	ZJ-1667
ZJ-1664(H6N1)	256	256
ZJ-1667(H6N1)	256	256
A/chicken/Zhejiang/727031/2014(H6N2)	16	16
A/duck/Zhejiang/727038/2014(H6N2)	16	16
A/chicken/Zhejiang/727001/2014(H6N6)	32	32
A/chicken/Zhejiang/727028/2014(H6N6)	16	16
A/chicken/ Zhejiang/110102/2015(H6N6)	16	16
A/chicken/Zhejiang/C1/2013(H9N2)	2	2
A/goose/Zhejiang/112071/2014(H5N1)	4	4

### Viral growth kinetics

**Fig. 3:**
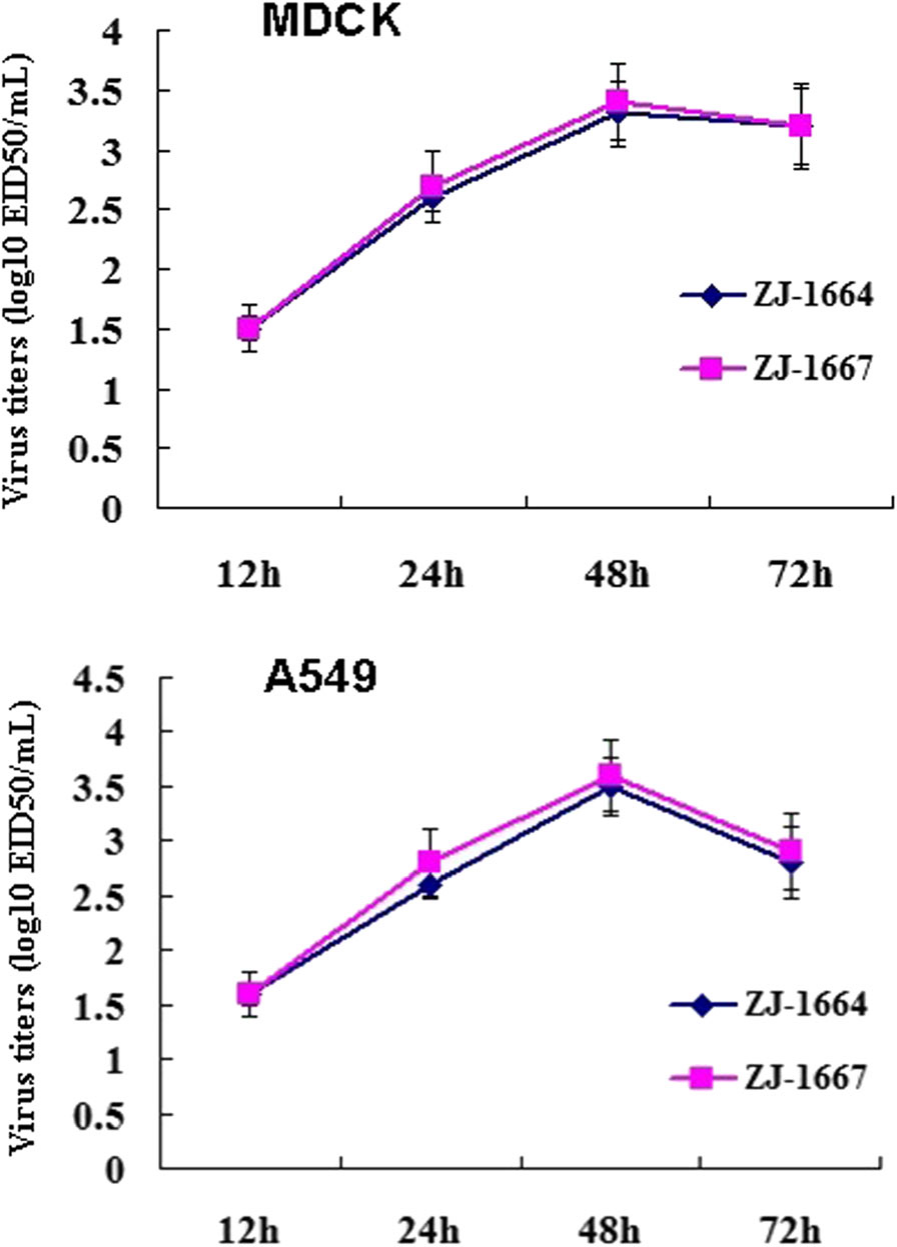
In vitro growth properties of the H6N1 avian influenza viruses. Growth curves were determined for each virus in MDCK or A549 cells after inoculation at an MOI of 0.1. Each point on the curve indicates the mean and standard deviation from three independent experiments

The in vitro growth properties of the ZJ-1664 and ZJ-1667 viruses were characterized in MDCK and A549 cells. The growth curves in MDCK and A549 cells showed that both viruses reached a maximum at 48 postinoculation, and suggesting that they have good replication capacity in mammalian cells (Fig. 3).

### Pathogenicity in mice

**Fig. 4:**
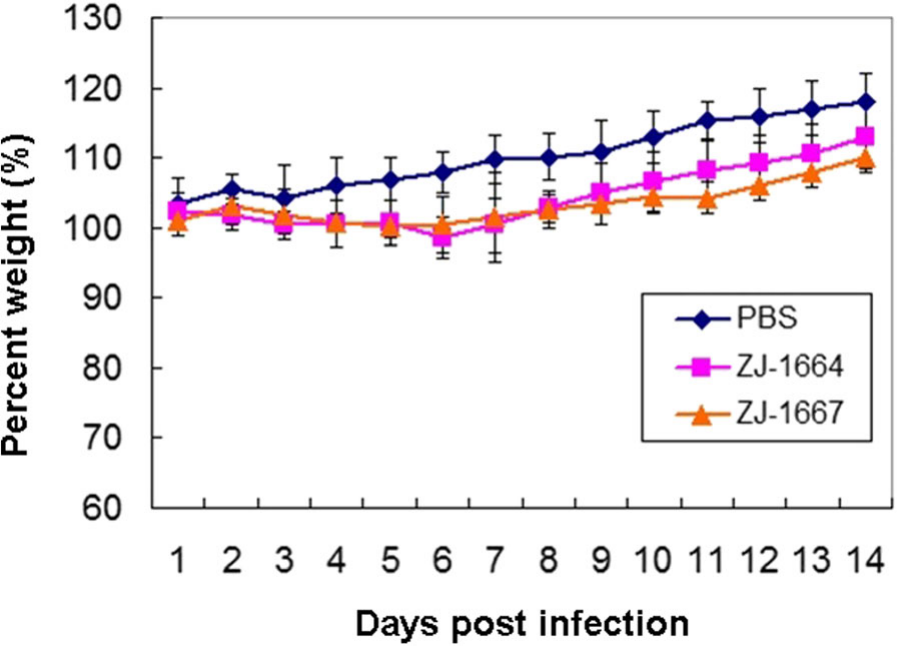
Weight variation of BALB/c mice infected with the H6N1 avian influenza viruses. Each mouse in a group was infected intranasally with 10^6.0^ EID50 of each virus (50 ul). A group inoculated with PBS (50 ul) as control was included. The body weight of mice were measured daily from the date of challenge to 14 days after challenge

To evaluate the pathogenicity and replicative potential of the ZJ-1664 and ZJ-1667 viruses in mammalian hosts, BALB/c mice were infected intranasally with 10^6.0^ EID_50_ of each virus. The mice infected with these two viruses exhibited slight weight-loss (no significant difference) (Fig. 4). These H6N1 viruses were able to replicate without prior adaptation. Six days after inoculation, high titers of ZJ-1664 and ZJ-1667 were detected in the lungs of infected mice, and could not be detected in the brain, spleen, liver, or kidney of infected mice. Additionally, six days after inoculation ZJ-1664 could also be detected in cardiac tissue from infected mice. Survival rates 14-days post-inoculation were 100% (6/6; Table 5).

**Table 5:** Mammalian viral replication of the novel reassortant H6N1 avian influenza viruses isolated from poultry in Eastern China, ZJ-1664 and ZJ-1667

Virus	No. of survivors/no. tested	HI titer (log2)	Virus replication in experimentally infected mice Virus titers in organs of mice (log_10_ EID_50/_ml)			
			Tissue	3 day	6 day	9 day
ZJ-1664	6/6	6.33 ± 0.52	Lung	2.67 ± 0.57	3.67 ± 0.57	2.33 ± 0.57
			Brain	0	0	0
			Heart	0	1.33 ± 0.57	0
			Liver	0	0	0
			Kidney	0	0	0
			Spleen	0	0	0
ZJ-1667	6/6	5.83 ± 0.75	Lung	2.33 ± 0.57	2.67 ± 0.57	1.33 ± 0.57
			Brain	0	0	0
			Heart	0	0	0
			Liver	0	0	0
			Kidney	0	0	0
			Spleen	0	0	0

Fifteen mice were intranasaly inoculated with 10^6.0^ EID50 of the H6N1 virus in a 0.05-ml volume of PBS. Six mice were observed for survival for 14 days. The remaining nine mice from each experimental group were exsanguinated on day 3, 6 and 9, and lungs, brains, hearts, kidneys, spleens, and livers were collected for virus titration in embryonated chicken eggs by the method of Reed and Muench. Sera were harvested at 14 days after infection from the six remaining mice. Seroconversion was confirmed by the hemagglutination inhibition (HI) test. Data represent mean ± SD

**Fig. 5:**
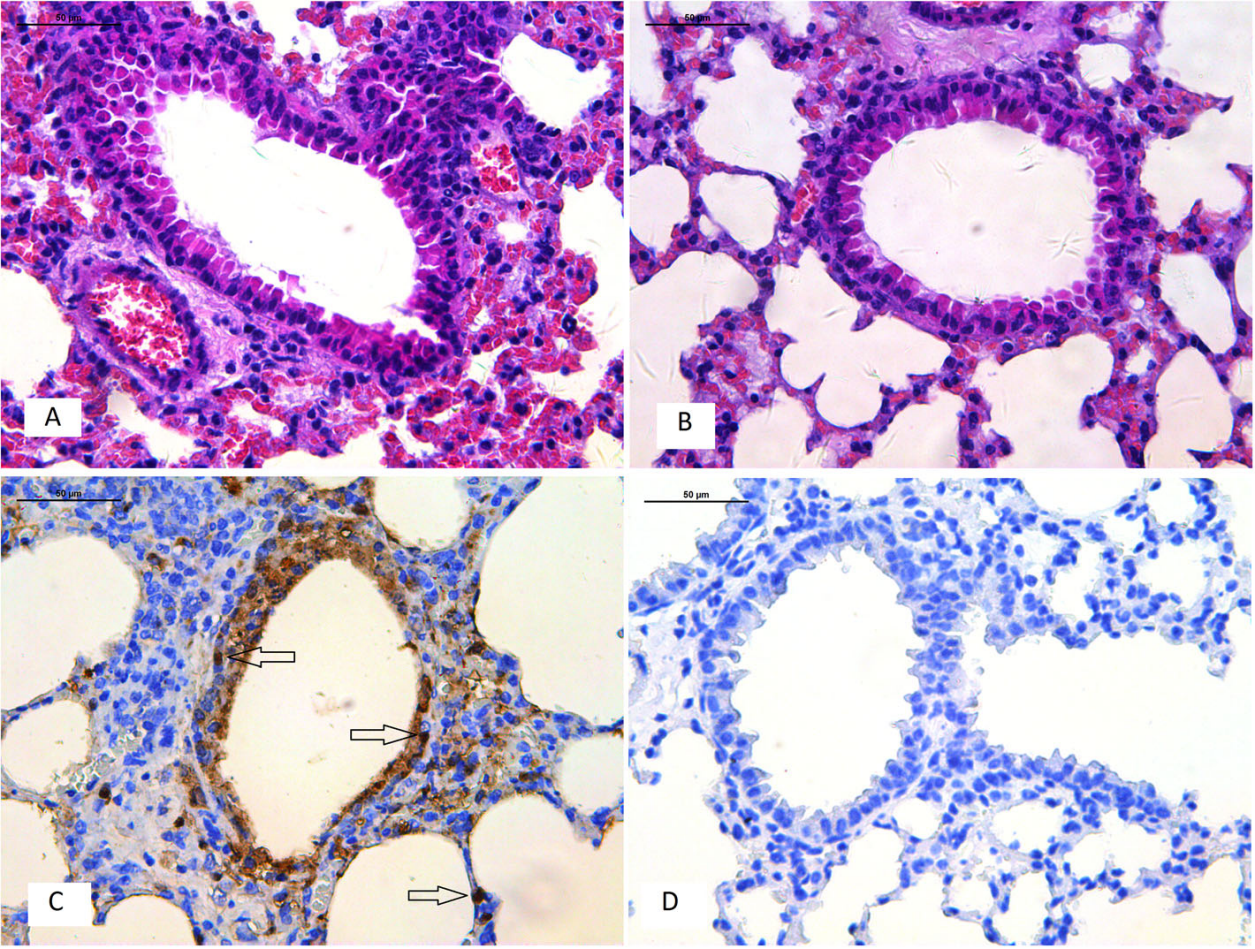
Histology and immunohistochemistry of mice infected with ZJ-1664 at 6 days post–infection. Histology of lung sections stained by hematoxylin and eosin from inoculated mice (**a**) or negative control (**b**). Immunohistochemical detection of virus nucleoprotein in lungs from inoculated mice (**c**) or negative control (**d**). Arrows indicate positively stained lung alveolar epithelial cells

Histopathological analyses showed that 6 days post–inoculation lung tissue had a light interstitial inflammatory hyperaemia (Fig. 5a). Immunohistochemistry was also performed to assess whether H6N1 viruses infected cells were present in tissues (including bronchial epithelial cells and alveolar epithelial cells) from infected mice 6 days post–inoculation (Fig. 5b). The H6N1 viruses were able to replicate in both bronchial epithelial cells and alveolar epithelial cells.

## Discussion

In recent years, human infections with the H5 and H9 subtypes of AIVs have received considerable attention, and the H6 subtype of AIVs has gained little increase in awareness. In 1965, the H6 influenza viruses were first identified in turkeys in the United States; during the past 20 years, H6 viruses have become established in poultry populations across Asia and have been isolated from poultry worldwide [14, 16–18, 26, 55]. In the United States, the zoonotic transmission of H6 viruses to humans has also been suggested by reports of significantly elevated titers of H6-specific antibodies among veterinarians routinely exposed to avian species [56]. A systematic serologic study showed that 0.4% serum specimens (63/15,689) of workers in the poultry industry in mainland China were positive for the H6 virus [20]. Recently, H6 viruses have been detected in mammalian species, such as dogs and pigs, in Asian countries [23, 57]. Many previous studies have demonstrated that several subtypes of H6 influenza viruses (H6N1, H6N2, H6N5, H6N6 and H6N8) are cocirculated in domestic duck and chicken populations in China, which provide the opportunity for significant viral reassortment [14, 15, 18, 19, 26, 58, 59]. The prevalence of the H6 influenza viruses throughout the world, the ability of H6 strains to infect mammals, and the potential for future reassortment and variation raises concern about the pandemic potential of H6 AIVs.

On 20 May 2013, the world’s first human-infected case of H6N1 influenza viruses was reported in Taiwan [60]. Although Tzarum et al.’s research showed that this strain retained specificity for avian receptor [46], and demonstrates that the H6 viruses possess the ability to infect mammals [23, 24]. The persistent exposure of H6N1 viruses into human populations and the lack of pre-existing immunity to H6 viruses suggest the possible emergence of a pandemic human influenza virus [16, 17, 22].

Mice are often used as a mammalian model to study the pathogenesis of influenza A viruses, including avian strains [61, 62]. Many studies have reported variable pathogenicity of H6 strains in mice [18, 19, 63]. Specific amino acid substitutions in the PB2 (E627K), PA (T97I), and HA (N394 T) proteins of H6N1 viruses contribute to enhanced growth characteristics and altered cell tropism, and may also enhance the virulence H6N1 AIVs in mice [64, 65]. We did not observe any of these specific substitutions in the ZJ-1664 or ZJ-1667 H6N1 strains. Our results indicate that these novel H6N1 AIVs possess specificity toward both the avian and human receptors, which are in agreement to the results of chicken H6N1 HA reported in previous studies [25, 66]; and while they did not cause lethal infections in mice, they are able to replicate in the lungs of the mice without prior adaptation. It has been reported that 34% of the H6 viruses coming from Chinese poultry are able to bind human type receptors, and some of these strains are able to transmit in guinea pigs through direct inoculation or contact [16]. The continued circulation of H6N1 viruses may represent a threat to human health.

Wild birds are a natural reservoir for AIVs; AIVs can replicate efficiently in their natural hosts, but replicate poorly in other species. H6 viruses are established in poultry populations throughout China, and domestic ducks are considered a population that can perpetuate most AIVs. Because infected ducks generally do not show obvious clinical symptoms, they can act as intermediate hosts between migratory birds and terrestrial poultry in the AIV ecosystem in China [14, 15, 67]. Terrestrial poultry (such as chickens) have molecular characteristics suitable to be intermediate hosts for transmission of AIVs to humans, and such transmission could generate new influenza viruses with pandemic potential [6–8].

## Conclusions

In this study, we identified two novel H6N1 AIV strains, resulting from a reassortment event that occurred between the H6 AIV and other AIV subtypes, including the H10, H1, and H4 strains. Chickens from LPMs in Eastern China may play an important role in expanding the host range for AIVs. These strains were able to replicate in mice without prior adaptation. Considering that the reassorted H6N1 viruses were isolated from chickens in this study, it is possible that chickens play an important role as a permissive host in the reassortment of novel H6N1 AIVs. These results indicate continual reassortment between AIVs from different avian species, and continued circulation of these viruses may pose a potential threat to humans.

## Additional file

Additional file 1: **Figure S1.** Comparison of deduced HA amino acid sequences of H6 viruses using the BioEdit program. The P186L, E190V and G228S substitutions (H3 numbering system) are shown in boxes. (JPG 241 kb)

## Abbreviations

AIVs: Avian influenza viruses
EID50: 50% embryo infectious dose
HA: Hemagglutinin
LPMs: Live poultry markets
M: Matrix protein
MOI: Multiplicity of infection
NA: Neuraminidase
NP: Nucleocapsid protein
NS: Nonstructural protein
PA: Polymerase acidic protein
PB1: Polymerase basic protein 1
PB2: Polymerase basic protein 2
RBCs: Red blood cells
