# Supplementary figures and images for: Isolation and characterization of novel reassortant H6N1 avian influenza viruses from chickens in Eastern China
Source: Virol J. 2018 Oct 24;15:164. doi: 10.1186/s12985-018-1063-y (PMC6201551; doi:10.1186/s12985-018-1063-y)

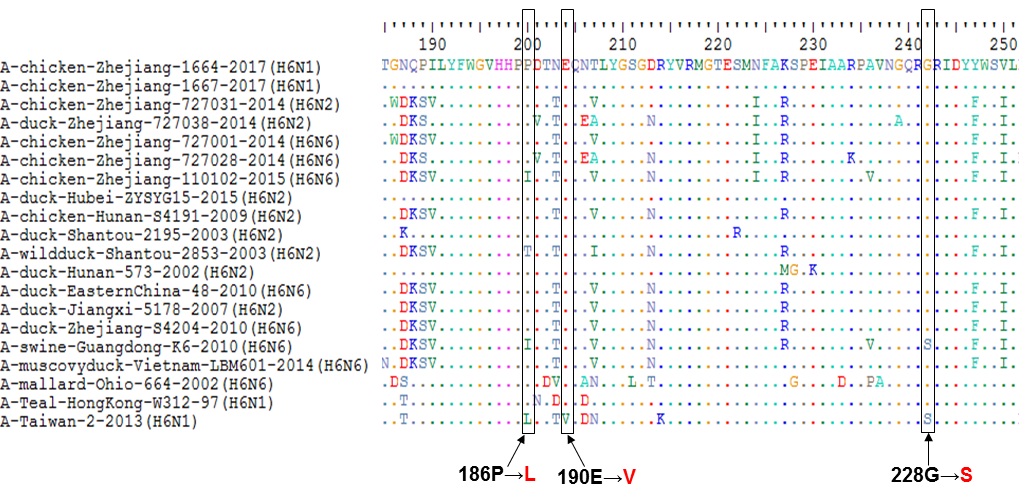

Supplement: Supplementary file 1 — Figure S1. Comparison of deduced HA amino acid sequences of H6 viruses using the BioEdit program. The P186L, E190V and G228S substitutions (H3 numbering system) are shown in boxes. (JPG 241 kb) [file 12985_2018_1063_MOESM1_ESM.jpg]
